# Synthesis of Multifunctional Hyperbranched Polymers via Atom Transfer Radical Self-Condensing Vinyl Polymerization for Applications in Polyurethane-Based Anion Exchange Membranes

**DOI:** 10.3390/polym17141930

**Published:** 2025-07-13

**Authors:** Nhat Hong Nguyen, Chih-Feng Huang, Tongsai Jamnongkan

**Affiliations:** 1 Department of Chemical Engineering, i-Center for Advanced Science and Technology (iCAST), National Chung Hsing University, Taichung 40227, Taiwan; nguyenhongnhat1510@gmail.com; 2 Graduate Program in Semiconductor and Green Technology, Academy of Circular Economy, National Chung Hsing University, Nantou City 540216, Nantou County, Taiwan; 3 Department of Fundamental Science and Physical Education, Faculty of Science at Sriracha, Kasetsart University, Chonburi 20230, Thailand

**Keywords:** hyperbranched polymers, ATR-SCVP, polyurethane, AEM

## Abstract

Anion exchange membranes (AEMs) are vital for electrochemical energy devices such as alkaline fuel cells and water electrolyzers, enabling the use of non-precious metal catalysts despite challenges from alkaline degradation. Hyperbranched polymers (hbPs) with their globular structure, high functional group density, and simple synthesis, offer a promising platform for enhancing transport and stability. In this study, multifunctional hbPs were synthesized from 4-vinylbenzyl chloride (VBC) and 2-hydroxyethyl methacrylate (HEMA) via atom transfer radical self-condensing vinyl polymerization (ATR-SCVP) and crosslinked into polyurethane-based AEMs. Characterization confirmed successful copolymerization and crosslinking, with excellent alkaline stability. Membranes crosslinked with higher molecular weight (MW) and VBC-richer hbPs (e.g., OH-hbP1-PU) exhibited high water uptake (75%) but low ion-exchange capacity (1.54 mmol/g) and conductivity (186 µS/cm), attributed to steric hindrance and insufficient ionic network connectivity. In contrast, OH-hbP2-PU exhibited optimal properties, with the highest OH^−^ conductivity (338 µS/cm) and IEC (2.64 mmol/g), highlighting a balanced structure for efficient ion transport. This work offers a tunable strategy for high-performance AEM development through tailored hbP architecture.

## 1. Introduction

Anion exchange membranes (AEMs) have garnered considerable attention for their promising role in electrochemical energy systems, such as alkaline fuel cells (AFCs), water electrolyzers, and direct ammonia fuel cells [1,2,3,4,5,6]. Compared with proton exchange membranes, AEMs operate under alkaline conditions, which enables the use of precious group metal (PGM) free catalysts, thereby lowering system costs and improving fuel flexibility [7,8,9]. However, the development of high-performance AEMs remains challenging due to the harsh alkaline environment, which often causes degradation of the polymer backbone and quaternary ammonium functional groups through mechanisms such as Hofmann elimination and nucleophilic substitution [10,11].

Recent research efforts have focused on designing robust polymeric backbones and optimizing microphase-separated structures to enhance both chemical stability and ionic conductivity [12,13,14,15,16,17,18]. Among various architectures, hyperbranched polymers (hbPs) are particularly appealing due to their globular topology, high density of functional end groups, internal free volume, and easy synthesis from commercially available monomers [19,20,21,22]. Owing to their unique properties, hbPs have been explored in a variety of applications including nanotechnology, biomedicine, composites, and adhesives [23,24,25]. These features also facilitate ion transport and improve water retention and dimensional stability under hydrated conditions. For instance, hbPs based on poly(aryl ether), poly(aryl piperidinium), and poly(styrene) have demonstrated outstanding ion conductivity and alkaline durability [19,20,21,26,27,28,29]. However, controlling the branching density, hydrophilic/hydrophobic balance, and mechanical integrity of these polymers remains a critical challenge for practical membrane fabrication.

To meet these design requirements, various synthetic approaches have been developed to construct hyperbranched architectures. Traditional methods, such as step-growth polymerization with AB_x_-type monomers, offer synthetic simplicity but often suffer from limited control over molecular weight and dispersity [30,31]. More advanced strategies based on controlled/living polymerization (CLPs) mechanisms, including self-condensing ring-opening polymerization [32], proton transfer polymerization [33], and controlled radical polymerization (CRP) techniques [34,35,36], have been developed. Several CLP-based branching methods, such as ATRP, RAFT, NMP, and ROP [37,38,39,40], have been reported. Except for the ATRP-based branching method, the synthesis of most inimers using the other CLP-based branching methods is tedious and cumbersome [41]. Thus, atom transfer radical self-condensing vinyl polymerization (ATR-SCVP) has emerged as a particularly versatile approach, enabling the one-pot synthesis of well-defined hbPs with tunable branching density, molecular weight control, narrow polydispersity, and high functional group accessibility [42,43,44]. For example, the molecular weights of hbPs can be controlled through conversion, reaction time, inimer-to-common monomer ratios, and degree of branching [45]. The ability of ATR-SCVP to copolymerize various vinyl monomers under mild conditions further highlights its suitability for constructing multifunctional hyperbranched polymers for advanced membrane applications.

**Scheme 1:**
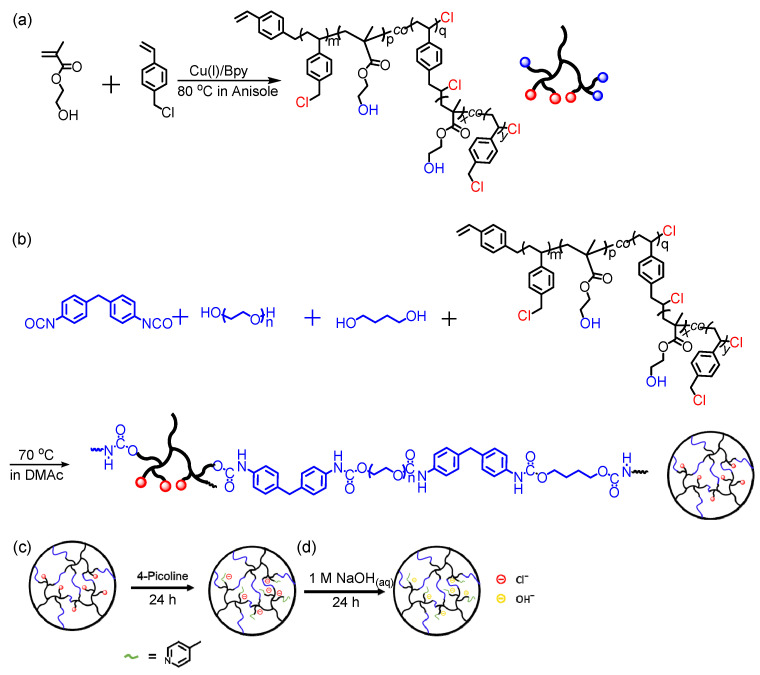
Consecutive preparations of (**a**) hbP(VBC-*co*-HEMA) via ATR-SCVP, (**b**) hbP(VBC-*co*-HEMA)-PU crosslinked with polyols, (**c**) Q-hbP(VBC-*co*-HEMA)-PU quaternized with 4-picoline, and (**d**) OH-hbP(VBC-*co*-HEMA)-PU ion-exchanged with NaOH_(aq)_.

In this study, we report the synthesis of multifunctional hyperbranched copolymers based on commercially available monomers of 4-vinylbenzyl chloride (VBC) and 2-hydroxyethyl methacrylate (HEMA) via atom transfer radical self-condensing vinyl polymerization (ATR-SCVP), as illustrated in Scheme 1. VBC serves as both a branching unit and a source of chloromethyl groups for quaternization, whereas HEMA introduces hydrophilic hydroxyl and ester moieties that can participate in polyurethane crosslinking and enhance water affinity. These copolymers were further crosslinked with diisocyanate to fabricate polyurethane-based AEMs. The resulting membranes were comprehensively characterized to evaluate their water uptake, swelling ratio, ion-exchange capacity (IEC), OH^−^ conductivity, and alkaline stability. Importantly, we demonstrate that increasing the density of hydrophilic and ionizable sites through hyperbranched architecture can significantly enhance IEC and water uptake without compromising membrane morphology, thereby overcoming the common trade-offs observed in conventional AEM systems. This approach offers a versatile platform for designing next-generation AEMs with well-balanced ionic and mechanical performance under alkaline conditions.

## 2. Materials and Methods

### 2.1. Materials

4-Vinylbenzene chloride (VBC, 98%), 4-picoline (98%), and 2-hydroxyethyl methacrylate (HEMA, 97%) were purchased from Acros (Geel, Belgium) and purified by passing through a column filled with basic alumina to remove the inhibitor. Polyethylene glycol 2000 (PEG2000) and copper(I) bromide (CuBr, 98%) were purchased from Thermo Fisher Scientific (Waltham, MA, USA). Copper(II) bromide (CuBr_2_, 98%) was purchased from Alfa Aesar (Ward Hill, MA, USA). 4,4′-Methylenebis(phenyl isocyanate) (MDI, 99%), 1,4-butanediol (BDO, 98%), 2,2′-bipyridine (Bpy, 99%) were purchased from Sigma-Aldrich (St. Louis, MI, USA). NaOH (98%) and HCl_(aq)_ (1 M) were purchased from Fluka (Buchs, Switzerland). CuBr was purified by washing with glacial acetic acid, filtered, washed with anhydrous ethanol, and drying under vacuum. All solvents were distilled before use.

### 2.2. Synthesis of Hyperbranched Poly(VBC-co-HEMA) (hbP(VBC-co-HEMA))

Various molar ratios of VBC/HEMA were mixed with Bpy (0.114 g, 0.75 mmol), CuBr_2_ (0.006 g, 0.025 mmol), and anisole in a round-bottom flask. After three freeze-pump-thaw cycles of degassing, CuBr (0.018 g, 0.25 mmol) was added to the reactor. Oxygen was removed by a few additional freeze-pump-thaw cycles. The reactor was placed in an oil bath at 80 °C, and the reaction rate was monitored by gas chromatography (GC). To terminate the reaction, the mixture was cooled and diluted with tetrahydrofuran (THF) to dissolve the polymer and oxidize the catalyst. The solution was filtered through an alumina column to remove the insoluble copper complex. The crude product was dissolved in THF and reprecipitated in hexane to remove the unreacted monomer. Finally, the solid was collected and placed in a vacuum oven overnight, resulting in a white solid.

### 2.3. Preparation of hbP-Crosslinked PU Membranes (hbP(VBC-co-HEMA)-PU)

PEG2000 diol (0.7 g, 0.35 mmol), hbP(VBC-*co*-HEMA) polyol, and 10 mL DMAc were added to a two-neck round-bottom flask and heated to 70 °C in an inert atmosphere. MDI (0.35 g, 1.4 mmol) was gradually added to the flask over 30 min. Chain extender of BDO (0.63 mL, 0.7 mmol) (NCO/OH = 1:1) was then added, and the mixture was further reacted for 1.5 h. The resulting viscous solution was cast into an aluminum foil mold and placed in an oven at 60 °C for 24 h of post-curing. After drying and removing it from the mold, a crosslinked and uniform membrane of hbP(VBC-*co*-HEMA)-PU was obtained (thickness = about 60 μm).

### 2.4. Preparation of Quaternized hbP(VBC-co-HEMA)-PU (Q-hbP(VBC-co-HEMA)-PU) and Ion-Exchanged (OH-hbP(VBC-co-HEMA)-PU) Membranes

The hbP(VBC-*co*-HEMA)-PU crosslinked membranes were immersed in a 10 vol.% 4-picoline/toluene at 50 °C overnight to perform quaternization between the benzyl chloride and pyridine groups via the Menshutkin reaction. The membranes were extensively rinsed multiple times with deionized water (DIW) to afford Q-hbP(VBC-*co*-HEMA)-PU membranes. These were then soaked in a 1 N NaOH_(aq)_ solution for 24 h to conduct ion exchanges from Cl^−^ to OH^−^. These membranes were neutralized by washing with DIW several times to produce OH-hbP(VBC-*co*-HEMA)-PU membranes, which can be further applied to serve as anion-exchange membranes (AEMs).

### 2.5. Characterization

The structure of hbP(VBC-*co*-HEMA) was determined by ^1^H NMR (Varian Unity INOVA 400 MHz (Palo Alto, CA, USA)) using chloroform-*d* (CDCl_3_) as the solvent. Fourier transform infrared (FT-IR) spectra were collected using a PerkinElmer (Waltham, MA, USA) Spectrum One spectrometer with 64 scans at a resolution of 1 cm^−1^. Powder samples were finely ground with KBr and compressed into pellets using a hydraulic press. Membrane samples were directly analyzed in the transmission mode without the need for additional preparation. The VBC conversion was monitored by GC using the solvent as an internal standard. Analyses were performed on a Hewlett–Packard (Palo Alto, CA, USA) 5890 Series II gas chromatograph equipped with a flame ionization detector (FID) and a CNW CD-5 capillary column. The molecular weight (e.g., *M*_n_ and *M*_w_) and molecular weight dispersity index (*Đ*_M_ = *M*_w_/*M*_n_) of the resulting hbP(VBC-*co*-HEMA) samples were determined by GPC in THF at a flow rate of 1.0 mL/min and 40 °C, using a Waters (Milford, MA, USA) 410 differential refractometer and PSS SDV columns of Linear S and 100 Å pore sizes. Calibration was performed using the PSt standard. Thermal stability was assessed by thermogravimetric analysis (TGA) using a Q50 analyzer (TA Instruments (New Castle, DE, USA)) operated under nitrogen from 25 to 700 °C at a heating rate of 20 °C/min. Differential scanning calorimetry (DSC) measurements were conducted on a Q20 calorimeter (TA Instruments (New Castle, DE, USA)) under a nitrogen atmosphere. The mechanical properties were evaluated using a Shimadzu (Kyoto, Japan) AGS-X STD universal testing machine. A dumbbell-shaped mold was used to prepare samples for the tensile test with dimensions of WxL = 3 × 60 (mm^2^) and a membrane thickness approximately 50 µm. Tensile tests were performed at a stretching rate of 5 mm/min at room temperature.

### 2.6. Water Uptake (W.U.) and Swelling Ratio (S.R.)

Water uptake (W.U.) and swelling ratio (S.R.) were used as the water absorption measurements. They affect the ion exchange capacity and mechanical stability [29]. The membranes were dried in a vacuum oven at 80 °C to obtain W_dry_. The membranes were immersed in DIW for 24 h to obtain W_wet_. W.U. and S.R. were calculated by the following Equations (1) and (2), respectively.

W.U. (%) = (W_wet_ − W_dry_)/W_wet_ × 100% (1)

S.R. = (W_wet_ − W_dry_)/W_dry_ (2)

### 2.7. Ionic Conductivity (IC)

Ionic conductivity (IC, mS/cm) was calculated by electrochemical impedance spectroscopy (EIS) [46]. The AEM in OH^−^ form was covered with two stainless steel sheets. The testing environment was maintained at 100% humidity, and the AEMs were immersed in DIW. The Metrohm (Herisau, Switzerland) Autolab PGSTAT302N was used for measurement at a temperature of 80 °C and the ionic resistance was obtained. The ionic conductivity was determined using Equation (3).

σ = L/RA(3)

Here, L (cm) is the distance between the working and counter electrode; A (cm^2^) is the cross-sectional area; R (Ω) is the Ohm impedance obtained from the electrochemical impedance spectra.

### 2.8. Ion Exchange Capacity (IEC)

The ion exchange capacity (IEC, mmol/g) exhibits the number of exchangeable ions per membrane dry weight (W_dry_) [47]. The IEC of the AEMs was measured using the acid/base titration method. The membrane in OH^−^ form was weighed and soaked in 1 N HCl_(aq)_ to convert the AEM to Cl^−^ form. After 24 h, the AEM was removed and rinsed with DIW. The resulting diluted HCl_(aq)_ solution was titrated with 1 N NaOH_(aq)_ solution using phenolphthalein as an indicator. The IEC was calculated using the following equation.

IEC = (V_acid_ × C_acid_ − V_base_ × C_base_)/W_dry_ (4)

## 3. Results and Discussion

**Figure 1:**
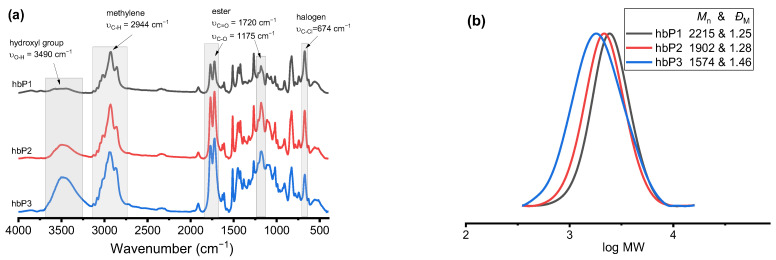
(**a**) FTIR spectra and (**b**) GPC traces of hbP(VBC-*co*-HEMA) samples.

The hbP(VBC-*co*-HEMA) polymers were synthesized with varying VBC and HEMA feed ratios, as summarized in Table 1. As illustrated in Figure 1a, their chemical structures were characterized by IR spectroscopy. The presence of broad hydroxyl (-OH) stretching at 3490 cm^−1^ and halogen stretching at 674 cm^−1^ confirmed the formation of multifunctional polymers. Notably, the intensity of the hydroxyl peak increased progressively from hbP1 to hbP3, which is correlated with the increased HEMA content. In addition, the characteristic ester signals of HEMA were identified at 1720 cm^−1^ (C=O) and 1175 cm^−1^ (C–O), respectively. Furthermore, the stretching vibration of the methylene groups at 2944 cm^−1^ supported the formation of a hyperbranched polymer architecture. Figure 1b shows the *M*_n,GPC_ and *Đ*_M_ determined by GPC. The highest *M*_n,GPC_ was observed for hbP1, followed by hbP2 and hbP3. This decreasing trend was correlated with the reduced VBC feed ratio, as VBCs act as both a vinyl monomer and an inimer in the ATR-SCVP process. A higher VBC content leads to more initiating sites and branching points, facilitating the formation of larger hyperbranched macromolecules. In contrast, a lower VBC ratio limits chain growth and branching, resulting in reduced molecular weight.

**Figure 2:**
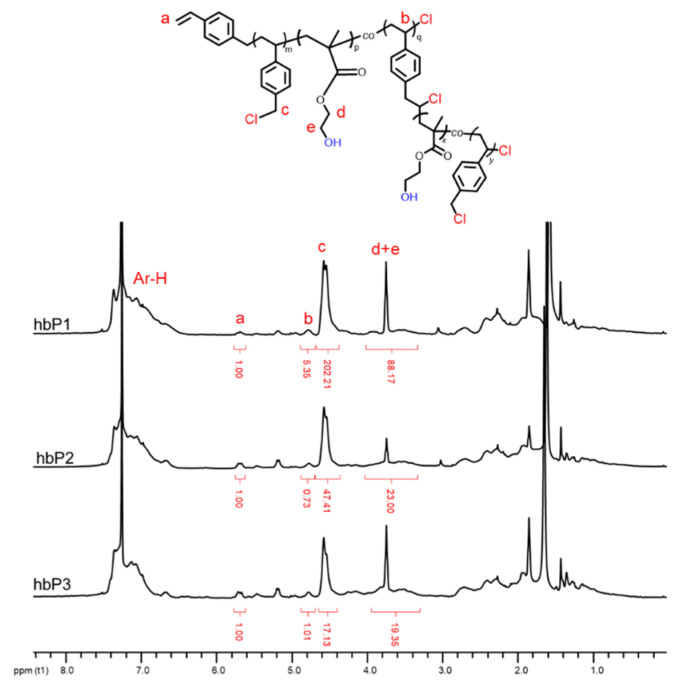
^1^H NMR (400 MHz, CDCl_3_) of hbP(VBC-*co*-HEMA) samples.

Figure 2 shows the chemical structure of hbP(VBC-*co*-HEMA) analyzed by ^1^H NMR spectroscopy. The characteristic signals corresponding to the VBC repeating units were observed in the range of δ = 4.2–4.9 ppm, which can be attributed to the benzylic -CH_2_- groups and -CH- groups adjacent to the chloride functional group. The aromatic protons of the VBC moiety appeared between δ = 7.0–7.4 ppm, consistent with the phenyl ring structure. A gradual decrease in the relative intensity of VBC-related signals was observed between hbP1 and hbP3, consistent with the reduced VBC feed ratio. Methyl protons of the methacrylate group were detected at δ = 1.85 ppm. Meanwhile, the methylene protons from the ethylene glycol side chain appeared as broad multiplets in the δ = 3.2–4.0 ppm range. These include both -CH_2_- groups adjacent to the ester oxygen and the terminal hydroxyl group. The presence and integration of these signals confirm the successful incorporation of HEMA and reflect the flexible, hydrophilic nature of its side chain, which plays a crucial role in tuning the physical properties of the resulting hyperbranched polymer.

The quantitative NMR compositional analysis results in Table 1 reveal that increasing the feed ratio of HEMA resulted in higher incorporation in the final polymer. The HEMA molar fraction increased from 16% in hbP1 to 34% in hbP3. This compositional variation is correlated with a significant decrease in the molecular weight. The absolute number-average molecular weights were characterized by NMR (*M*_n,NMR_), which indicated decreasing to 18,910 g/mol for hbP1 and to 2091 g/mol for hbP3. A similar trend was observed for *M*_n,GPC_, although the absolute values were systematically lower due to calibration with linear PSt standards. This reduction in molecular weight can be explained by the reduced number of branching points because VBC units serve as multifunctional branching sites enabling hyperbranching growth, whereas HEMA predominantly acts as a monofunctional monomer, limiting network extension during SCVP. Furthermore, *Đ*_M_ slightly increased from 1.25 to 1.46 with HEMA content, reflecting broader molecular weight distributions likely caused by irregular incorporation and heterogeneous branching. More importantly, the branching index (BI) increased markedly from 0.12 to 0.75 as the VBC content decreased from hbP1 to hbP3, indicating a structural transformation toward less compact, more linear polymer chains.

**Figure 3:**
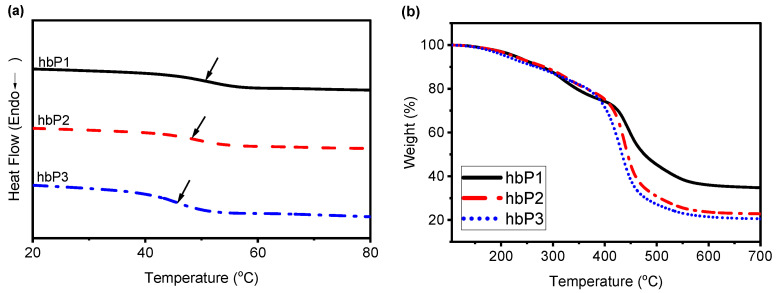
(**a**) DSC traces and (**b**) TGA profiles of hbP(VBC-*co*-HEMA) samples.

The thermal properties of hbP(VBC-*co*-HEMA) samples are presented in Figure 3 and summarized in Table 2. As shown in Figure 3a, the glass transition temperature (*T*_g_) exhibited a gradual decline from 51 °C for hbP1 to 45 °C for hbP3. This trend can be attributed to the increased incorporation of HEMA, which introduces more flexible ethylene glycol segments into the polymer backbone. The reduced rigidity of the network, resulting from the lower aromatic content and fewer rigid benzyl branches provided by VBCs, contributes to enhanced chain mobility and thus lowers the *T*_g_ of the system. The thermal degradation profiles further demonstrate the influence of the monomer composition on the polymer stability. The temperature at 5 wt% decomposition (*T*_d5%_) of hbP1 reached 226 °C, reflecting its higher aromatic content and greater degree of branching, both of which enhanced thermal resistance. In contrast, hbP2 and hbP3 exhibited lower degradation temperatures, consistent with their reduced VBC content and more linear architectures. Moreover, the residual mass at high temperature decreased from hbP1 to hbP3, suggesting that the reduced presence of thermally stable aromatic rings and halogenated groups from VBC leads to a less char-forming structure upon degradation. These findings confirm that increasing HEMA content, while beneficial for flexibility and functionality, compromises thermal robustness due to a less crosslinked and more aliphatic rich polymer framework.

**Figure 4:**
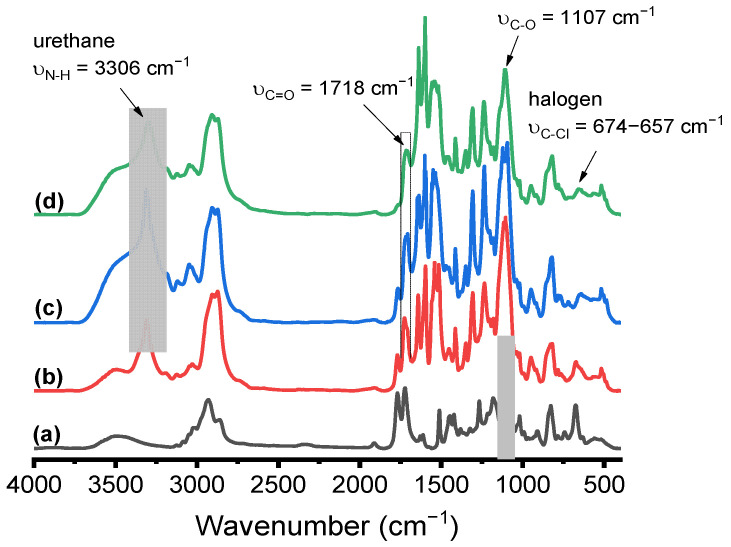
Exemplary IR spectra of (**a**) hbP2, (**b**) hbP2-PU membrane, (**c**) Q-hbP2-PU membrane and (**d**) OH-hbP2-PU membrane.

Exemplary structural evolutions of the membranes after each treatment are shown in Figure 4b,c (i.e., hbP2-PU and Q-hbP2-PU membranes, respectively). The successful quaternization of hbP2-PU membranes with 4-picoline was confirmed by the significant decrease in the intensity of the C–Cl stretching band at 674 cm^−1^, which also shifted slightly to 657 cm^−1^. This shift reflects the conversion of benzylic chloride groups to quaternary ammonium sites, indicating efficient ionization. Furthermore, new IR absorption spectra were obtained after the formation of the polyurethane membrane. Specifically, a characteristic N–H stretching peak of the urethane linkage appeared at 3306 cm^−1^, while the absence of a signal at 2257 cm^−1^ confirmed that no residual isocyanate groups remained, indicating a complete reaction during the polyurethane network formation. Additionally, the carbonyl (C=O) stretching band at 1718 cm^−1^ and the C–O stretching at 1107 cm^−1^ indicated the presence of urethane and ester functionalities in the polymer backbone. Moreover, to evaluate the chemical robustness of the membrane under alkaline conditions, the FTIR spectra were recorded after immersion in 1 N NaOH_(aq)_ solution. As illustrated in Figure 4d (i.e., OH-hbP2-PU membrane), no significant spectral changes were observed, indicating that the chemical structure was intact. This result highlights the excellent alkaline stability of the membrane, which is an essential criterion for AEM applications operating under strongly basic conditions.

The thermal stability of the synthesized membranes was evaluated by TGA, and the results are summarized in Table S1 (see Supplementary Materials). A comparison of the thermal degradation profiles of the membranes in their OH^−^ form is presented in Figure S1 (see the Supplementary Materials). Among the series, OH-hbP3-PU exhibited the highest early-stage thermal stability, with decomposition temperatures of *T*_d5%_ and *T*_d10%_ recorded at 279 and 307 °C, respectively. This was followed by OH-hbP1-PU and OH-hbP2-PU in descending order. This thermal performance of OH-hbP3-PU is likely attributed to its relatively high HEMA content, which contributes to increased crosslinking density through urethane formation, thereby enhancing the thermal resistance of the membrane. However, considering the temperature at 20% weight loss and the final residual mass, OH-hbP1-PU demonstrated superior high-temperature stability, showing the greatest char yield of 29.5%. This trend reflects the influence of BI and *M*_n_, both of which are highest for hbP1. The more hyperbranched architecture of OH-hbP1-PU contributes to enhanced thermal rigidity and restricted chain mobility, resulting in slower degradation in the later stages and the formation of more stable carbonaceous residues. In contrast, despite showing high initial resistance to decomposition, OH-hbP3-PU may undergo more extensive bond cleavage at elevated temperatures due to its lower branching density and ester group thermal lability. These findings highlight the dual role of hyperbranched structure in affecting both the onset and extent of thermal degradation in polyurethane-based AEMs. To further investigate the effect of chemical modifications on thermal behavior, TGA was also conducted on the membranes before quaternization, after quaternization with 4-picoline, and following OH^−^ ion exchange, as shown in Figure S2 (see Supplementary Materials). A marked decrease in both *T*_d5%_ and *T*_d10%_ was observed after quaternization. This reduction may be due to the incorporation of thermally less stable quaternary ammonium groups, which undergo degradation under elevated temperatures. Interestingly, the thermal stability was improved after hydroxide ion exchange. This enhancement could be attributed to the ionic interactions within the hydrated matrix and the possible reorganization of polymer chains, which delay the onset of thermal decomposition.

Tensile testing was conducted to evaluate the mechanical performance of the AEMs. The results are summarized in Table S2 and illustrated in Figure S3 (see Supplementary Materials). The tensile strength progressively increased from OH-hbP1-PU to OH-hbP3-PU, reaching a maximum value of 205.4 MPa for OH-hbP3-PU. This enhancement can be attributed to the higher HEMA content and more linear molecular architecture in OH-hbP3-PU, which likely promotes better packing and stronger intermolecular interactions within the polyurethane matrix. Furthermore, OH-hbP3-PU exhibited the highest Young’s modulus of 46,590 kN/m^2^, indicating its superior stiffness and elastic deformation resistance under load. In contrast, the highest elongation at break of 22.9% was observed for OH-hbP2-PU, suggesting that it possesses the greatest ductility among the samples. This may be due to its moderately branched structure, which balances chain flexibility and network cohesion. Meanwhile, OH-hbP1-PU and OH-hbP3-PU exhibited lower tensile strains of 7.8% and 7.1%, respectively, reflecting their more rigid or tightly packed architectures. Despite its lower modulus, OH-hbP2-PU also demonstrated superior toughness (14,416 kJ/m^3^), allowing it to undergo greater plastic deformation while maintaining structural integrity. These findings highlight the critical influence of monomer composition and hyperbranched architecture on the mechanical behavior. While OH-hbP3-PU offers higher strength and stiffness due to dense packing and high crosslink density, OH-hbP2-PU provides enhanced flexibility and energy absorption capacity, making it a promising candidate for applications where mechanical resilience under dynamic conditions is essential.

**Figure 5:**
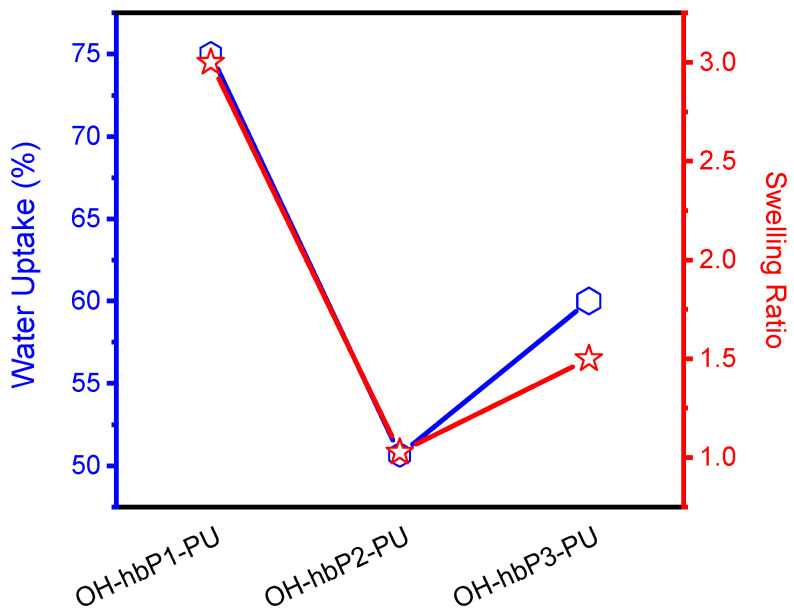
Water uptake and swelling ratio of OH-hbP(VBC-*co*-HEMA)-PU membranes (pentagon: water uptake; star: swelling ratio).

Figure 5 illustrates the water uptake (WU) and swelling ratio (SR) of hbP(VBC-*co*-HEMA)-PU membranes in their OH^−^ form. Interestingly, OH-hbP1-PU synthesized from hbP1 with the lowest HEMA ratio exhibited the highest WU (75%) and SR (3.0). This unexpected behavior can be explained by considering higher *M*_n_ values. The elevated *M*_n_ of hbP1 indicates a more hyperbranched and less crosslinked network in the final AEM, which facilitates more free volume and water-accessible pathways, thereby promoting a higher water uptake and swelling ratio. In contrast, the OH-hbP2-PU derived from hbP2 exhibited the lowest water uptake (50%) and swelling ratio (1.0). Meanwhile, OH-hbP3-PU synthesized from hbP3 showed slightly higher values of 60% and 1.5, respectively. Although hbP3 contains more HEMA units, which are inherently hydrophilic, its higher BI value indicates a more linear architecture than hbP2. Consequently, OH-hbP3-PU forms a less crosslinked and more flexible network, allowing greater water diffusion and dimensional expansion. These results indicate that the membrane hydration behavior is governed not only by the hydrophilicity introduced by the HEMA content but also by the degree of branching and molecular weight of the precursor polymers.

**Figure 6:**
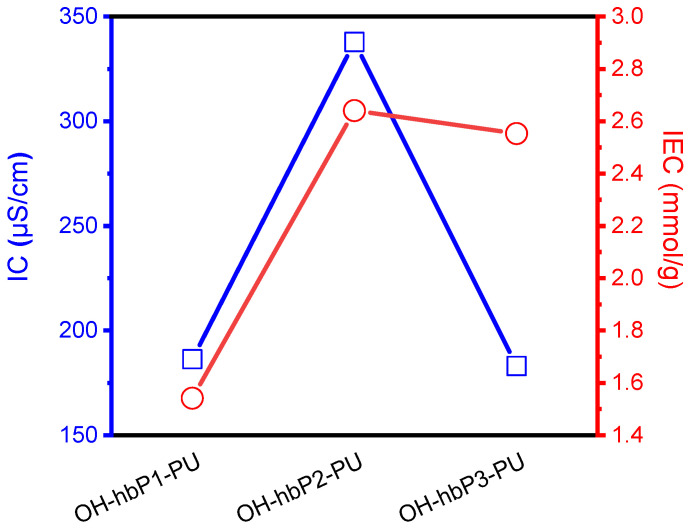
OH^−^ conductivity and ion-exchange capacity of OH-hbP(VBC-*co*-HEMA)-PU membranes (square: IC; circle: IEC).

Hydroxide ion conductivity and ion exchange capacity (IEC) are crucial parameters for evaluating AEM performance. As shown in Figure 6, OH-hbP2-PU achieved the highest OH^−^ conductivity (338 μS/cm) and IEC (2.64 mmol/g), indicating the most efficient ion-transport characteristics among the tested membranes. On the other hand, OH-hbP1-PU exhibited the lowest IEC (1.54 mmol/g) and conductivity (186 μS/cm). This unexpected result can be explained by considering the structural and morphological effects of hyperbranching. Although hbP1 possesses the highest VBC feed ratio, which provides a greater theoretical number of chloromethyl groups for quaternization, the resulting membrane (OH-hbP1-PU) exhibits the lowest IEC and conductivity. This can be attributed to the highly branched architecture, which may lead to steric hindrance during quaternization, limiting the accessibility of some benzyl chloride sites with 4-picoline. As a result, not all available -CH_2_Cl groups are effectively converted into ion-exchangeable quaternary ammonium sites. Moreover, the excessively branched structure of OH-hbP1-PU can cause inefficient ionic domain connectivity, impairing OH^−^ transport despite its high water uptake. The significant swelling observed in OH-hbP1-PU may further dilute the local ionic concentration, thereby reducing the effective conductivity. Similarly, OH-hbP3-PU synthesized from hbP3 exhibited a higher IEC (2.55 mmol/g) than OH-hbP1-PU, but only a slightly higher conductivity (183 μS/cm). This may be attributed to its more linear structure, which, as previously discussed, hampers the formation of continuous ionic pathways despite having more ionizable groups. These observations highlight that high conductivity in AEMs requires a high IEC and a well-balanced polymer architecture that enables effective ion channel formation without excessive swelling or dilution effects.

## 4. Conclusions

This study demonstrates the successful synthesis of multifunctional hyperbranched copolymers from VBC and HEMA via ATR-SCVP. The resulting copolymers were crosslinked with diisocyanate to form polyurethane-based AEMs. Structural characterization using FTIR and ^1^H NMR confirmed the incorporation of both monomers. The VBC content significantly influenced the molecular weight and branching, whereas the HEMA content affected the thermal stability. The membranes exhibited excellent alkaline stability, which is essential for electrochemical energy applications such as fuel cells and water electrolyzers. The effect of polymer architecture on ion transport was a critical finding. Although OH-hbP1-PU had the highest VBC content, it showed the lowest ion-exchange capacity (IEC, 1.54 mmol/g) and OH^−^ conductivity (186 µS/cm) due to steric hindrance and poor ionic domain connectivity. In contrast, OH-hbP2-PU exhibited superior performance, with an IEC of 2.64 mmol/g and a conductivity of 338 µS/cm, indicating optimal branching for ion channel formation. These results offer a promising strategy for tuning hyperbranched polymer structures to develop high-performance AEMs that overcome common trade-offs under alkaline conditions.

## Figures and Tables

**Table 1:** Characterization of hbP(VBC-*co*-HEMA) samples.

Sample	Molar Feed Ratio		Product Ratio		*M*_n,NMR_	*M*_n,GPC_	*Đ*_M_	BI ^a^
	VBC	HEMA	VBC	HEMA				
hbP1	90	10	84	16	18,910	2215	1.25	0.12
hbP2	80	20	81	19	4431	1902	1.28	0.43
hbP3	70	30	66	34	2091	1574	1.46	0.75

^a^ Branching index = *M*_n,GPC_/*M*_n,NMR_ [44].

**Table 2:** Thermal properties of hbP(VBC-*co*-HEMA) samples.

Sample	*T*_g_ (°C)	*T*_d5%_ (°C)	*T*_d10%_ (°C)	Residual Mass (%)
hbP1	51.48	226.3	278.1	34
hbP2	49.49	224.1	283.5	22
hbP3	45.20	208.8	267.9	20

## Data Availability

The original contributions presented in this study are included in the article/Supplementary Materials. Further inquiries can be directed to the corresponding authors.

## Supplementary Materials

The following supporting information can be downloaded at: https://www.mdpi.com/article/10.3390/polym17141930/s1, Table S1: Thermal properties of hbP(VBC-*co*-HEMA)-PU, Q-hbP(VBC-*co*-HEMA)-PU, and OH-hbP(VBC-*co*-HEMA)-PU membranes; Table S2: Mechanical properties of OH-hbP(VBC-*co*-HEMA)-PU membranes; Figure S1: TGA profiles of OH-hbP(VBC-*co*-HEMA)-PU membranes; Figure S2: Comparisons of exemplary TGA profiles of hbP3-PU, Q-hbP3-PU, and OH-hbP3-PU membranes; Figure S3: Mechanical properties of OH-hbP(VBC-*co*-HEMA)-PU membranes.
