# Supplementary material for: Synthesis of Multifunctional Hyperbranched Polymers via Atom Transfer Radical Self-Condensing Vinyl Polymerization for Applications in Polyurethane-Based Anion Exchange Membranes
Source: Polymers (Basel). 2025 Jul 13;17(14):1930. doi: 10.3390/polym17141930 (PMC12298691; doi:10.3390/polym17141930)
Supplement: Supplementary file 1 [file polymers-17-01930-s001.zip › polymers-3741612-supplementary.pdf]

polymers

Supplementary Materials for

Synthesis of Multifunctional Hyperbranched Polymers via Atom Transfer Radical Self-Condensing Vinyl Polymerization for Applications in Polyurethane-Based Anion Exchange Membranes

Nhat Hong Nguyen [1], Chih-Feng Huang [1,2,*] and Tongsai Jamnongkan [3,*]

[1] Department of Chemical Engineering, i-Center for Advanced Science and Technology (iCAST), National Chung Hsing University, Taichung 40227, Taiwan; nguyenhongnhat1510@gmail.com
[2] Graduate Program in Semiconductor and Green Technology, Academy of Circular Economy, National Chung Hsing University, Nantou City 540216, Nantou County, Taiwan
[3] Department of Fundamental Science and Physical Education, Faculty of Science at Sriracha, Kasetsart University, Chonburi 20230, Thailand
* Correspondence: huangcf@dragon.nchu.edu.tw (C.-F.H.); jamnongkan.t@ku.ac.th (T.J.)

Captions.

Table S1. Thermal properties of hbP(VBC-*co*-HEMA)-PU, Q-hbP(VBC-*co*-HEMA)-PU, and OH-hbP(VBC-*co*-HEMA)-PU membranes.

Table S2. Mechanical properties of OH-hbP(VBC-*co*-HEMA)-PU membranes.

Figure S1. TGA profiles of OH-hbP(VBC-*co*-HEMA)-PU membranes.

Figure S2. Comparisons of exemplary TGA profiles of hbP3-PU, Q-hbP3-PU, and OH-hbP3-PU membranes.

Figure S3. Mechanical properties of OH-hbP(VBC-*co*-HEMA)-PU membranes.

Table S1. Thermal properties of hbP(VBC-*co*-HEMA)-PU, Q-hbP(VBC-*co*-HEMA)-PU, and OH-hbP(VBC-*co*-HEMA)-PU membranes.

Sample	$T_{d5\%}$ (°C)	$T_{d10\%}$ (°C)	Residual mass (%)
hbP1-PU	238.4	303.6	16.9
Q-hbP1-PU	199.9	219.8	14.4
OH-hbP1-PU	269.3	303.9	29.5
hbP2-PU	269.1	313.9	21.5
Q-hbP2-PU	205.6	235.1	12.4
OH-hbP2-PU	264.8	301.0	17.6
hbP3-PU	288.3	314.0	10.4
Q-hbP3-PU	234.9	300.1	17.5
OH-hbP3-PU	279.7	307.8	11.8

Table S2. Mechanical properties of OH-hbP(VBC-*co*-HEMA)-PU membranes.

Sample	Tensile strength (MPa)	Tensile strain (%)	Young's Modulus (kN/m^2)	Toughness (kJ/m^3)
OH-hbP1-PU	56.5	7.8	17608	3277
OH-hbP2-PU	77.3	22.9	13464	14416
OH-hbP3-PU	205.4	7.1	46590	10140

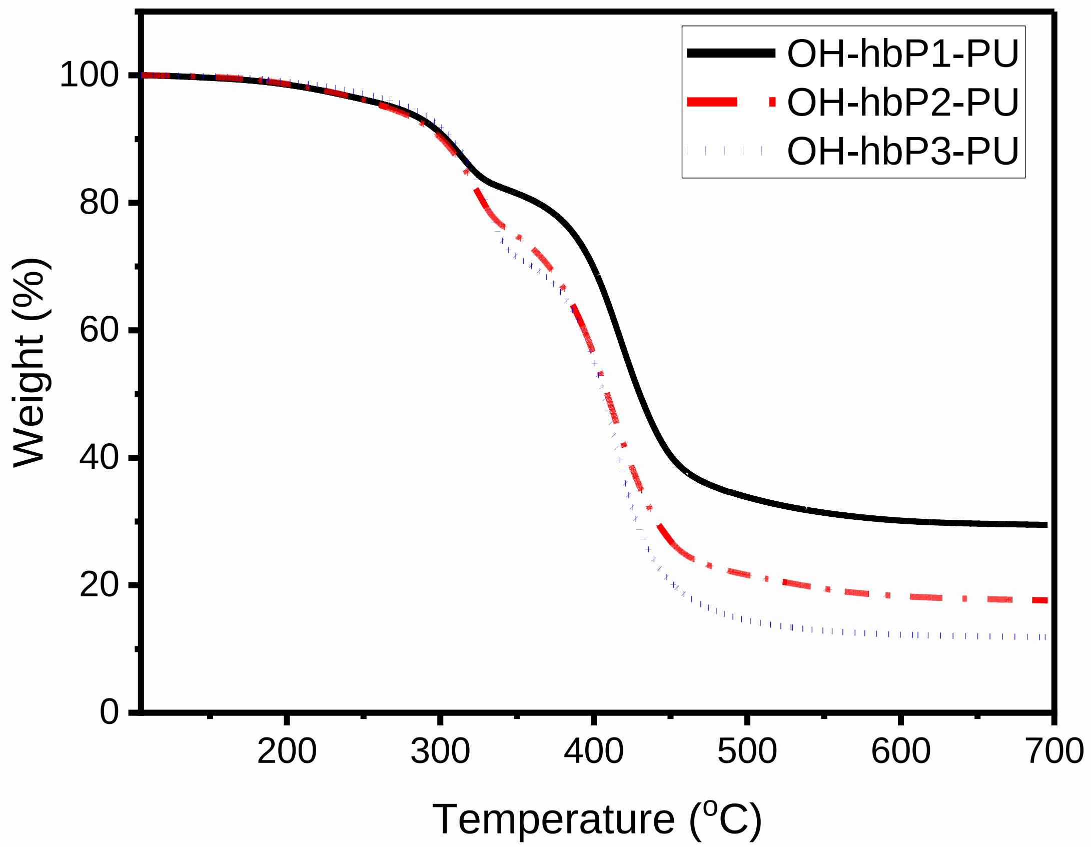

Figure S1. TGA profiles of OH-hbP(VBC-*co*-HEMA)-PU membranes.

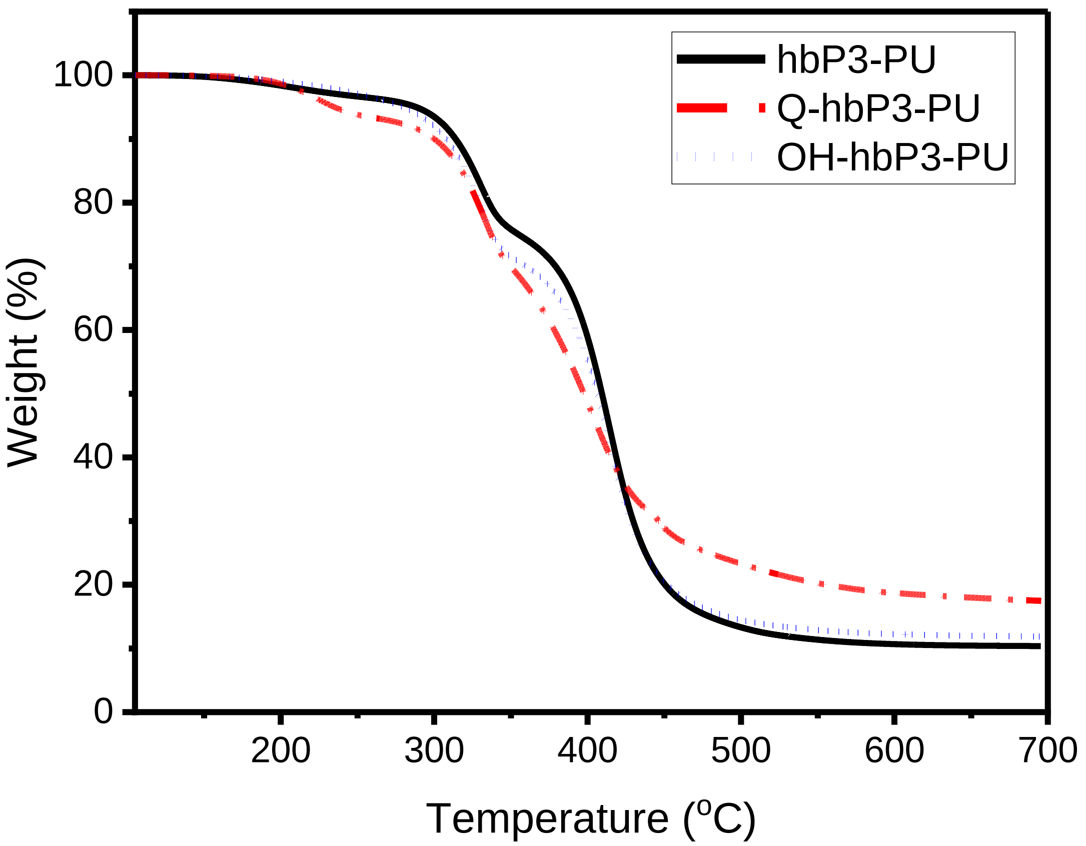

Figure S2. Comparisons of exemplary TGA profiles of hbP3-PU, Q-hbP3-PU, and OH-hbP3-PU membranes.

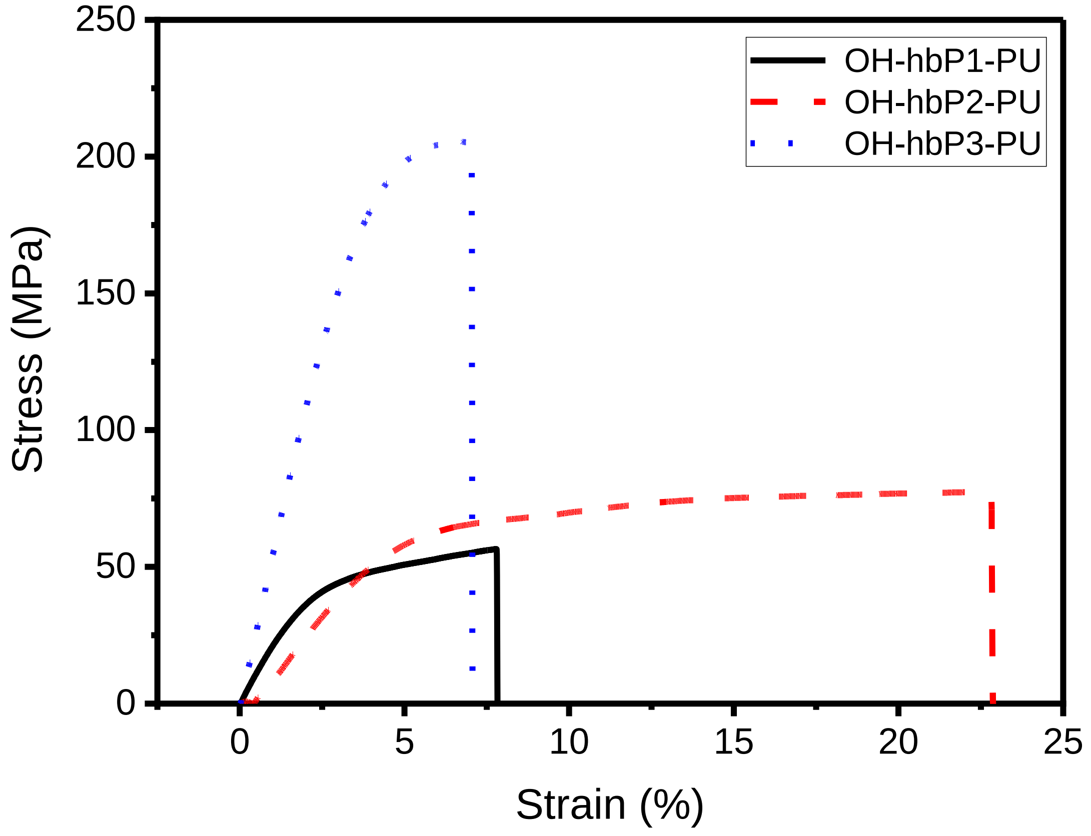

Figure S3. Mechanical properties of OH-hbP(VBC-*co*-HEMA)-PU membranes.
